# Formation of Highly Emissive Anthracene Excimers for Aggregation-Induced Emission/Self-Assembly Directed (Bio)imaging

**DOI:** 10.1021/acsami.3c10823

**Published:** 2023-09-12

**Authors:** Pedro J. Pacheco-Liñán, Carlos Alonso-Moreno, Alberto Ocaña, Consuelo Ripoll, Elena García-Gil, Andrés Garzón-Ruíz, Diego Herrera-Ochoa, Sofía Blas-Gómez, Boiko Cohen, Iván Bravo

**Affiliations:** † Unidad nanoDrug. Facultad de Farmacia de Albacete, Universidad de Castilla-La Mancha, 02008 Albacete, Spain; ‡ Experimental Therapeutics Unit, Hospital clínico San Carlos, IdISSC and CIBERONC, 28040 Madrid, Spain; § Unidad de Investigación del Complejo Hospitalario Universitario de Albacete. Oncología Traslacional, 02008 Albacete, Spain; ∥ Centro Regional de Investigaciones Biomédicas (CRIB), 02008 Albacete, Spain; ⊥ Centro de Innovación en Química Avanzada (ORFEO-CINQA), Universidad de Castilla-La Mancha, 02008 Albacete, Spain; # Departamento de Química Física, Facultad de Ciencias Ambientales y Bioquímica, and Instituto de Nanociencia, Nanotecnología y Materiales Moleculares (INAMOL), Universidad de Castilla-La Mancha, Avenida Carlos III, S/N, 45071 Toledo, Spain

**Keywords:** aggregation-induced emission, AIEgens, living systems, molecular rotors, breast cancer, fluorescence lifetime imaging microscopy, FLIM, T-shaped anthracene dimers

## Abstract

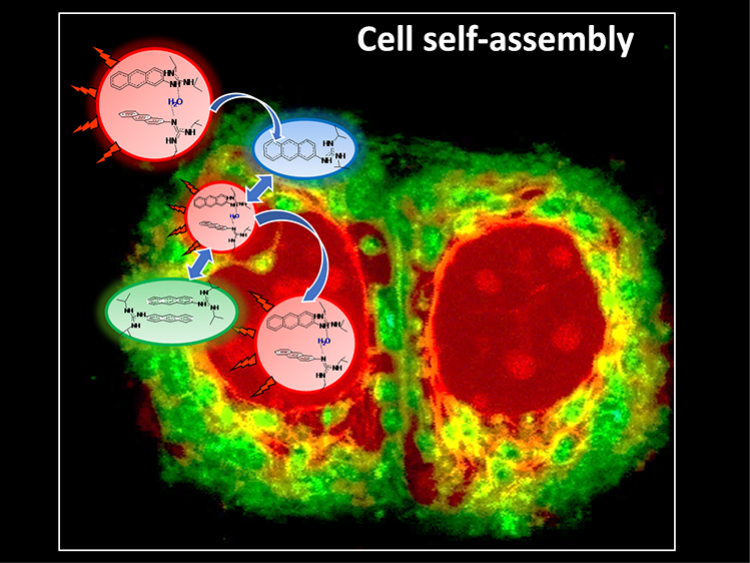

AIEgens have emerged as a promising alternative to molecular rotors in bioimaging applications. However, transferring the concept of aggregation-induced emission (AIE) from solution to living systems remains a challenge. Given the highly heterogeneous nature and the compartmentalization of the cell, different approaches are needed to control the self-assembly within the crowded intricate cellular environment. Herein, we report for the first time the self-assembly mechanism of an anthracene-guanidine derivative (AG) forming the rare and highly emissive T-shaped dimer in breast cancer cell lines as a proof of concept. This process is highly sensitive to the local environment in terms of polarity, viscosity, and/or water quantity that should enable the use of the AG as a fluorescence lifetime imaging biosensor for intracellular imaging of cellular structures and the monitoring of intracellular state parameters. Different populations of the monomer and T-shaped and π–π dimers were observed in the cell membrane, cytoplasm, and nucleoplasm, related to the local viscosity and presence of water. The T-shaped dimer is formed preferentially in the nucleus because of the higher density and viscosity compared to the cytoplasm. The present results should serve as a precursor for the development of new design strategies for molecular systems for a wide range of applications such as cell viscosity, density, or temperature sensing and imaging.

Fluorescence emission of molecular rotors is related to segmental mobility where rotation of subunits enables switchable molecular conformations and charge transport states transducing light energy into mechanical rotation. Any environmental restriction can either effectively quench or promote fluorescence. Such environmental sensitivity of fluorescent molecular rotors has been attractive for use in biomedical applications including bioimaging and diagnosis or local polarity and viscosity sensing. However, fast rotation in a molecular rotor requires a small energy barrier height, which disables its controllability, resulting in low environmental sensitivity and poor fluorescence intensity-contrast trade-off that limits practical performance in the biomedical field.^1−8^ To overcome such limitations, one of the emerging strategies due to its multifunctional potential is the use of fluorescent molecules with aggregation-induced emission characteristics (AIEgens).^9^

Several studies have demonstrated the applicability of AIEgens as contrast agents for fluorescence imaging with two fundamental underlying mechanisms for the enhanced emission of AIEgens forming the ground for their use in bioimaging. The first one is related to the restriction of the internal rotational motion of the AIEgen groups once bound with biomolecules or influenced by the surrounding bioenvironment, while the second one is related to the spontaneous aggregation under specific local environmental conditions.^10^ Of particular interest is the molecular self-assembly that plays a key role in many biological processes, like the formation of cell membranes, cell motion, and intracellular or extracellular transport of metabolites.^11−13^ An increasing number of molecules with adequate conformation that exhibit AIE have been developed with the aim to obtain functional materials for applications in nanotechnology and molecular optoelectronics.^14−16^ Such functional materials and molecular devices use controlled aggregation to form assemblies with synergistic properties that significantly differ from those of the constituent parts. Extensive efforts have been made in the development of such approaches for biomedical applications such as drug delivery, biosensing, tissue engineering, polymer therapeutics, and others.^2,17−21^ For example, pyrene-based AIE-active materials have been reported and were reviewed recently focusing on the AIE property toward sensing, imaging, and theranostic applications.^22,23^ However, transitioning the molecular self-assembly process from the solid state or solution to a living system still remains a challenge, as it requires precise control over the intermolecular forces involved, which often combine hydrophobic, electrostatic interactions and hydrogen bonds in complex environments. These intrinsic characteristics of AIEgens results in short wavelength absorption, broad emission, and aggregation-dependent brightness that indirectly may limit their practical performance in the biomedical field. However, AIEgen molecules for fluorescence lifetime imaging and sensing could overcome such inconveniences.^9^

Among the myriad of AIEgens, anthracene and its derivatives have been extensively investigated for applications in material science based on their excellent luminescence properties due to the formation of different supramolecular structures that range from linear dimers to π–π stacking arrangement of the anthracene units.^24−28^ Thus, several attempts have been made to use anthracene derivatives in the biomedical field, encompassing anticancer drug design, biosensing, cross-linkers, hybrid materials, and others.^29−35^ From all the possible supramolecular arrangements that anthracene and its derivatives may adopt, the unusual T-shaped geometry is the most sought after due to its excellent luminescence properties.^36−41^ However, its formation in a biological environment has never been described. We recently reported on a new anthracene-guanidine derivative (AG) that is able to form the rare T-shaped dimer in aqueous solution where the water molecules play a fundamental role in assisting the self-assembly process through H-bonds. The T-shaped dimer is present in the ground state and behaves as a highly emissive static excimer upon photoexcitation.^40,42,43^ The formation of this dimer in water was demonstrated in a previous work, showing an estimated size of 1.5 nm and where a water molecule acts as a bridge between two monomers.^40^ Thus, the T-shaped dimer formation is easy to control, and its stability depends mainly on the presence of external stimuli: pH values within 4–10, the water molar fraction ranging 0.8–1, and concentrations <10 μM reinforce the stability of the dimer and enhance its luminescence properties—an extremely high luminescence quantum yield (QY ≈ 1) and maximum emission at 515 nm along with a long fluorescence lifetime (≈25 ns).^40^ Other environmental stimuli such as ionic strength, viscosity, or polarity may also condition its formation along with π–π dimers/aggregates. In this work, we study for the first time the AIEgen mechanism of formation of T-shaped dimers in a biological environment. First, we start from the in vitro evaluation of stimuli responsiveness to later transfer to the biological system by employed breast cancer cell lines as a proof of concept. Then, we also assessed its environmental sensitivity to be used as a fluorescence lifetime imaging microscopy (FLIM) sensor for intracellular detection, including the fluorescence imaging of cellular structures (e.g., nucleus/nucleolus, membrane, etc.) and the qualitative monitoring of intracellular state parameters (e.g., viscosity, polarity, or water quantity).

## Materials and Methods

### Materials

Chemicals and samples used were SIB (Ficoll400 Sigma-Adrich, bovine serum albumin, Sigma-Aldrich, KCl ≥99.5% Labkem, NaCl ≥99.9% Labkem, CaCl_2_ ≥96% Sigma-Aldrich, MgSO_4_ 98% Merck, Bis-Tris Panreac 99%), glycerol (VWR chemicals), ethanol (≥99.8%, Sigma-Aldrich), and dimethylsulfoxide (DMSO, Sigma-Aldrich). Acetonitrile, tetrahydrofuran (THF), ethanol, *N*,*N*′-diisopropylcarbodiimide, amines, ZnEt_2_ (1 M solution in hexane), dichloromethane, and toluene were purchased from Sigma-Aldrich (Spain). Hexane, NaOH, and HCl were provided from Lubke (Spain). Deuterated water was purchased from Sigma-Aldrich (Spain). The highest purity grade available was used. All aqueous solutions were prepared in Milli-Q water and filtered with 0.22 μm filters prior to use. The pH of the aqueous solutions was adjusted using NaOH or HCl. Stock solutions were kept at 4 °C in the dark. Solvents were purified by passage through a column of activated alumina (Innovative Tech), degassed under nitrogen, and stored over molecular sieves in a glovebox prior to use.

### Synthesis of AG

The synthesis was prepared according to procedures reported in the literature.^40^ Briefly, 0.04 mL of a solution of ZnEt_2_ in hexane (1 M) was added to a solution of 1-aminoanthracene (2 mmol) in dry THF (20 mL) in a Schlenk tube. *N*,*N*′-Diisopropylcarbodiimide (2 mmol) was then added to the above reaction mixture. The Schlenk tube was taken outside the glovebox, and the reaction was carried out at 50 °C for 3 h. The solution was concentrated under reduced pressure, hexane was added, and the mixture was placed in a refrigerator at −30 °C for 16 h. After filtration, the guanidine products were obtained as white microcrystalline solids in 95% yield.

### Spectral Equipment and Measurements

Steady-state fluorescence (SSF) spectra were recorded on an FLS920 spectrofluorometer (Edinburgh Instruments) equipped with an MCP-PMT (microchannel plate-photomultiplier tube) detector (R3809 model) and a time-correlated single photon counting (TCSPC) data acquisition card (TCC900 model). A Xe lamp of 450 W was used as the light source for SSF spectra, and a sub-nanosecond pulsed light-emitting diode, EPLED-360 (Edinburgh Photonics), was employed as a light source at 368 nm for time-resolved fluorescence (TRF) decays. A TLC 50 temperature-controlled cuvette holder (Quantum Northwest) was used to keep the temperature at 35 °C during spectrum acquisition. Excitation and emission slits were both fixed at 4 nm, the step size was 1 nm and the dwell time was 0.1 s. The excitation wavelength (λ_ex_) was 368 nm, emission wavelength (λ_em_) was 520 nm, and the Δλ_em_ was 10 nm. All measurements were performed using a 10 mm quartz cuvette (Hellma Analytics).

Solutions of the AG (10 μM) were prepared in different solvents from stock solutions (1 mM) of the AG in the same solvent. For experiments in water, stock solutions of the AG (1 mM) in ethanol were used.

The fluorescence intensity decay, I(t), was fitted to the following multiexponential function using an iterative least-squares fit method
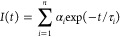
1where α_*i*_ and τ_*i*_ are the amplitude and lifetime for each *i*th term. The mean lifetime of the decay was then calculated as:
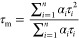
2

QYs were measured in an FS5 spectrofluorometer (Edinburgh Instruments) equipped with an integrating sphere, a 150 W Xe lamp as the light source, and a PMT (photomultiplier tube) detector (R928P model). QY calculations were carried out using F980 software of Edinburgh Instruments.

### Cell Cultures

Breast cancer cells, BT474 and MDA-MB-231, were grown in Dulbecco’s modified Eagle’s medium (DMEM) and MCF-7 in Eagle’s medium (EMEM). Each medium was supplemented with 10% inactivated fetal bovine serum, 2 mM l-glutamine, penicillin (20 units/mL), and streptomycin (5 μg/mL). Cell cultures were incubated at 37 °C in a saturated humidity atmosphere with 5% of CO_2_. For FLIM experiments, cells were seeded onto 20 mm square glass cover slides in a six-well plate and cultured (3 × 10^4^ cells per plate) at 37 °C in a 5% CO_2_ humidified atmosphere with their respective medium without phenol red. The cells were incubated with the AG (10 μM) for 1, 8, and 24 h. The cells were fixed with paraformaldehyde 10% for 15 min and washed with PBS. The samples were maintained in PBS at 4 °C until measure.

### MTT Metabolization Assays

For viability assessment of AG, cell proliferation was assayed by MTT (3-(4, 5-dimethylthiazol-2-yl)-2, 5 diphenyltetrazolium bromide) (Sigma-Aldrich). Cell lines were plated at 10,000 cells per well in 48-multiwell plates. After 24 h, the cells were treated at correspondent doses of the drugs for 72 h. After this, the medium was aspirated and phenol red-free DMEM with MTT 0.5 mg/mL was added for 60 min under growth conditions. The medium was removed and MTT crystals were solubilized with 0.5 mL of DMSO and evaluated at an absorbance of 555 nm in a multiwell plate reader.

### Dynamic Light Scattering

Size and polydispersity index of the AG and aggregates were analyzed by using the dynamic light scattering (DLS) technique on a Zetasizer Nano ZS instrument (Malvern Instruments). Data were analyzed using multimodal number distribution software included with the instrument.

### Fluorescence Lifetime Imaging of Cells

Fluorescence lifetime images were recorded with a MicroTime 200 microscope (PicoQuant) equipped with a TCSPC card and two TAU-SPAD-100 avalanche photodiode detectors. A 375 nm pulsed diode laser (LDH-D-C-375, PicoQuant) was used as the excitation source at a 10 MHz repetition rate and a power of ∼0.7 μW. The emission was recorded with a long-pass filter (−519/19 LP); 80 × 80 μm regions were scanned with 156 nm/pixel spatial resolution and 2 ms of dwell time. FLIM images were processed using SymPhoTime 64 software (PicoQuant). The lifetime distribution histograms were obtained from FLIM images and were fitted to the Gaussian curve. The emission spectra were collected through a Shamrock ST-303i (Andor Technology) imaging spectrograph and detected by an Andor Newton EMCCD camera (Andor Technology). For the spectral filtering experiments, we used Semrock BrightLine filters: FF01-445/40-25 and FF01-540/50-25. The FLIM images were smoothed over 200 nm for clarity of presentation. The emission spectra and the histograms were averaged over three independent measurements.

## Results and Discussion

### In Vitro Stimuli Responsive and Photodynamic Behavior

**Figure 1:**
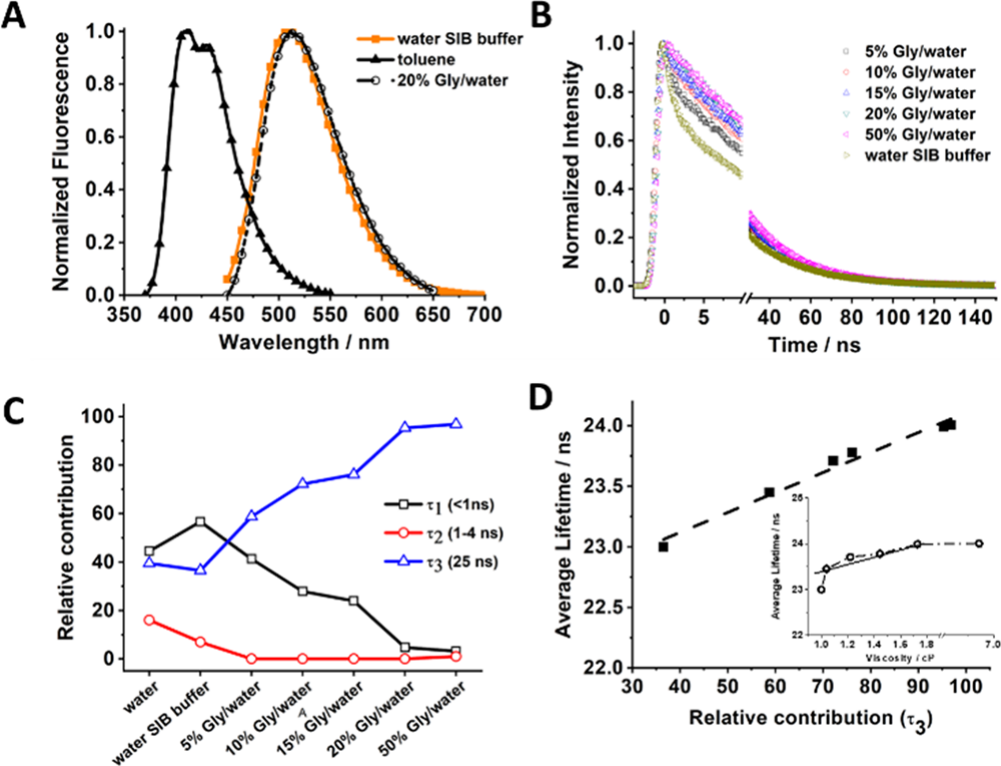
(A) Normalized emission spectra of the AG in different solvents. (B) Fluorescence decays of the AG in aqueous SIB pH 7.4 buffer and in the glycerol/water mixture (the arrow indicates increasing glycerol content). (C) Relative contribution (normalized to 100) of the observed fluorescence lifetimes to the overall fluorescence decay of the AG in different glycerol/water mixtures. (D) Average fluorescence lifetime values vs relative contribution of τ_3_ within SIB and glycerol/water mixtures (up to 50% v/v). Inset: average fluorescence lifetime vs the change of the viscosity.

To begin with, Figures 1A and S1A show the emission spectra of AG, while Figure S2 shows the measured average fluorescence lifetime in solvents with different polarities and viscosities. In toluene and dichloromethane, the emission spectra partially preserve the vibronic structure of the anthracene moiety, indicating that the predominant species in nonprotic and apolar solvents is the monomer form (maximum emission at 410–440 nm and fluorescence lifetime ≈11 ns).^40^ It should be noted that the reported fluorescence lifetime for the anthracene monomer is ∼5–6 ns.^44^ However, when the guanidine group is added, it gives rise to a significantly longer monomer lifetime due to the formation of charge separated states.^40^ Similar effects have been reported for other guanidine-functionalized organic chromophores, such as naphthalene and perylene.^45,46^ This assignment was further supported by previous concentration-dependent studies in toluene, where for significantly diluted AG solutions, the emission decayed monoexponentially with a time constant of ∼11 ns, but at increased concentrations, the emission decays showed the presence of additional population with a lifetime of ∼4 ns, assigned to formation of π–π dimers and aggregates.^40^ At high monomer concentrations, these dimers become larger π–π aggregates, which are less emissive, as verified herein by additional DLS experiments (Figure S1B). In acetonitrile, tetrahydrofuran, and ethanol, a red-shifted emission is observed (maximum emission within 490–500 nm along with lifetimes ranging between 11 and 15 ns, see Figures S1 and S2 related to the monomers and the possible formation of different populations of π–π dimers). Notably, we did not observe clear dependence both on the organic solvents’ viscosity and their polarity (Figure S2). We explain this behavior with the coexistence of at least two populations of the AG—the monomer (∼11 ns) and π–π dimes (∼4 ns)—that can present different sensitivity toward the solvent properties. However, and most importantly, no significant contribution of T-shaped dimer population (∼24 ns) was found in these media. On the other hand, it is possible that a trace amount of water in some of the used solvents can give rise to T-shaped dimer formation, hence increasing the value of the average fluorescence lifetime.

T-shaped dimer formation is observed exclusively in water and in aqueous synthetic intracellular 7.4 pH buffer (SIB) (maximum emission at 515 nm and an average lifetime of 23.1 ns), in agreement with a previous study that demonstrated the fundamental role that the water molecules play for its formation.^40^ Thus, it would be possible to control the formation of the different AG species by adjusting the water content and the solvent properties, with the T-shaped geometry achieved exclusively in aqueous solutions. Most notably, in the complex SIB that mimics the intracellular environment, containing proteins, polysaccharides, and high ion concentrations, the AG is also present as a T-shaped dimer. Additional 5 nm red-shifted emission is detected in a 20% (v/v) glycerol/water mixture, suggesting that the viscosity may further affect the T-shaped dimer formation and its stability by impeding in the more viscous solvents the molecular motion that might break the coupling between the anthracene units in the dimer.

To further explore the effect of viscosity, we recorded the emission spectra and fluorescence decays of the AG in different glycerol/water mixtures (0–50%; 1.0–6.8 cP). Figure 1B demonstrates a gradual change from multiexponential to monoexponential behavior with the increase of the glycerol content. This is concomitant with the small but notable red shift in the emission spectra (Figure S3). The relative contribution of the short components (τ_1_ < 1 ns and τ_2_ = 1–4 ns) associated with the presence of weakly emissive π–π dimers^40^ decreases with the increase in the glycerol content until becoming negligible for glycerol >20%. No component associated with the monomer (∼11 ns) is observed. On the other hand, the relative contribution of the long component (τ_3_ = 25 ns), which we assign to the T-shaped dimer, increases significantly and reaches 100% at higher glycerol contents (see Figure 1C). We found an excellent linear relationship between the obtained average lifetimes and the relative contribution of the longest component, τ_3_, upon increasing the glycerol content (Figure 1D). The higher contribution of τ_3_ results in a gradual growth of the average emission lifetimes from 23.1 ns in SIB to 24.0 ns in the 20–50% glycerol/water mixture associated with a change in the viscosity from 1 to 6.8 cP, respectively (inset of Figure 1D). This behavior suggests that the presence of water molecules, along with the increase in the viscosity of the environment, stabilizes the T-shaped species in a conformation that gives rise to an enhanced emission and longer average fluorescence lifetimes (Figure 1D). Such behavior is highlighted when the average emission lifetime of the AG is plotted against the glycerol molar fraction (χ_Gly_) where the maximum stabilization (higher average lifetime) is reached at χ_Gly_ = 0.06 corresponding to a 20% glycerol/water mixture. We found a similar trend for the fluorescence QY values (Figure S4 and Table S1). Note that at 50% glycerol (χ_Gly_ = 0.2), the value of the QY decreases significantly in comparison to the lower glycerol fraction. We explain this behavior with the significant restriction of the molecular motions in the highly viscous (∼7 cP) environment that impedes reaching optimal conformation of the T-shaped dimer. Additionally, this effect might be related to the reduced number of available water molecules that participate in the T-shaped dimer structure.

In summary, in organic solvents and in the absence of water molecules where the AG monomeric species and the weakly emissive π–π aggregates are predominant, no clear dependence on the solvent polarity and viscosity is observed and the respective average lifetimes vary between 10 and 15 ns. On the other hand, in aqueous solutions, the related lifetimes increase gradually with the viscosity of the surrounding media from ∼23.1 ns in SIB (because of the mixed contribution of the monomer, π–π dimers, and T-shaped dimer populations) to 24 ns when the glycerol addition increases the viscosity from 1.0 to 6.8 cP with predominant T-dimer contribution. Within the 1.0–1.8 cP viscosity range (Figure 1D), we found a linear relationship with the average fluorescence lifetime, which then becomes independent of the change of the viscosity for values >1.8 (inset of Figure 1D).

### FLIM Bioimaging and Sensing

Next, to explore the possible application of the intrinsic interplay between the photophysical properties of the different emissive populations of AGs, we studied their behavior in biological systems, where different physiological factors such as ionic strength, available water quantity, the presence of biological macromolecules, and the viscosity, among others, may affect their rate of formation or persistence. Fluorescence lifetime imaging confocal microscopy (FLIM) was used to monitor the population distribution of the AG in human breast cancer cell lines (MCF7, BT474, and MDA-MB231). MTT assays were also performed on the three cell lines, concluding that the AG does not cause toxicity with IC_50_ values >400 μM in all cell lines (Figure S5).

**Figure 2:**
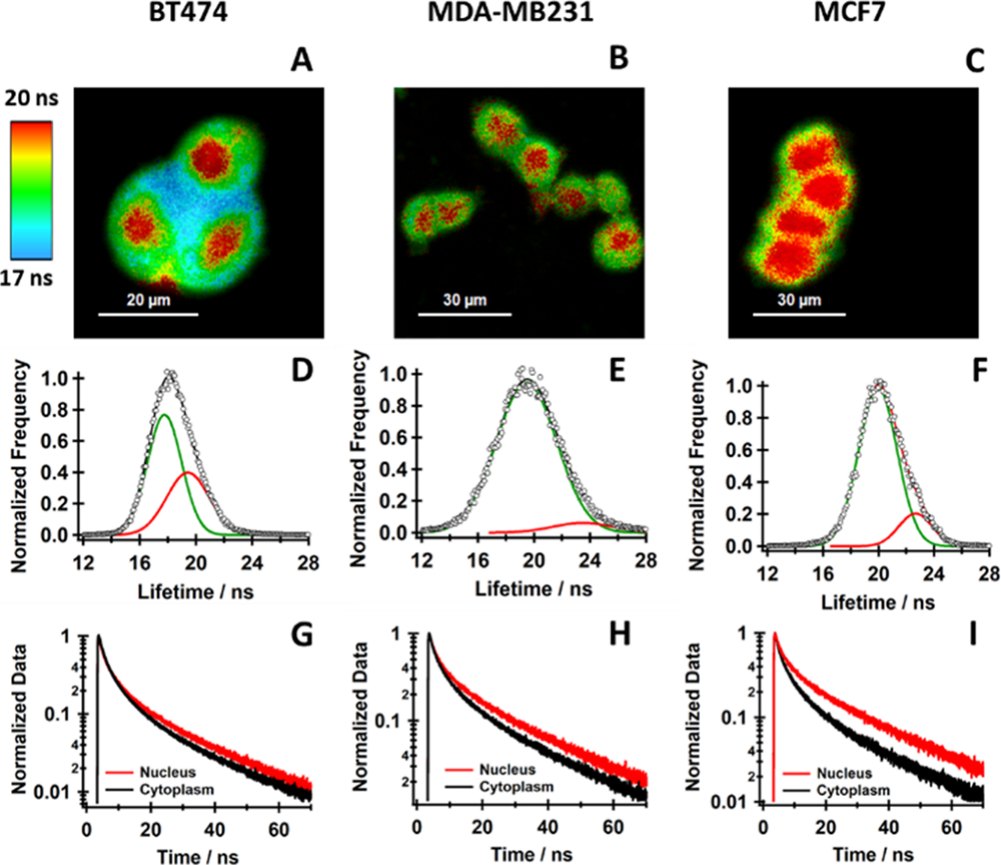
FLIM images of the studied cell lines; BT474 (A), MDA-MB231 (B), and MCF7 (C) along with the respective overall histograms of the average emission lifetimes (D– F) and the emission decays (G, H, and I) collected at selected points in the cell nucleus (red) and the cytoplasm (black). The scale bars for the FLIM images are 20 μm (A), 30 μm (B), and 30 μm (C).

The FLIM images of all the cell lines following an 8 h incubation (Figure 2 and Table S2) demonstrate a clear difference in the formation of the T-shaped dimer and the monomer/π–π dimer populations. To begin with, the overall average lifetime distributions for all the cell lines are broad and span from 12 to 27 ns. For the BT474 cells, the histogram is shifted to lower values with the maximum centered on 17.8 ns, whereas for MDA-MB231 and MCF7, the histograms are centered on 19.5 and 20 ns, respectively. Detailed analysis of the lifetime distribution histograms requires two Gaussian functions for an accurate fit, which suggests that the AG is present in different forms (monomer and different dimers) in the cell environment. Further analysis shows that the population with average lifetimes longer than 18 ns is located preferentially in the cell nucleus, while the one with lower lifetime values (<18 ns) is found in the cytoplasm. The deconvolution of the histograms for MDA-MB231 and MCF7 cell lines shows that the dominant contribution arises from a population of AG molecules with lifetimes of 19–20 ns. On the other hand, the maximum value of the lifetime for the second population is significantly different, ∼23 ns for both MDA-MB231 and MCF7. In the case of BT474, the deconvolution produces two distributions with close maximum lifetime values—17.8 ns (66%) and 19.5 ns (34%).

Next, we analyzed the lifetime distributions in the nucleoplasm and cytoplasm separately for all the cell lines. In all the FLIM images, it results in single Gaussian histograms (Figure S6 and Table S3). For BT474, the one for the cytoplasm is centered on 17.8 ns, while those for MCF7 and MDA-MB231 are located at 19.7 and 19.2 ns, respectively. The distributions for the nucleus are shifted to higher lifetime values, centered on 19.6, 22.8, and 23.6 ns for BT474, MCF7, and MDA-MB231, respectively. As observed in the emission decays of the AG in SIB and in the 50% glycerol/water mixture, the T-shaped dimer has a lifetime of 23–25 ns. Therefore, significantly larger lifetime values are associated to the nucleoplasm when compared to the cytoplasm for all the studied cell lines, suggesting higher viscosity in the former. It should be noted that average lifetime values lower than 20 ns correspond to environments with lower viscosity, polarity, and low water content (vide supra) where the AG is present predominantly as a monomer and π–π dimers. On the contrary, in aqueous solutions with higher viscosity, the AG is almost entirely converted to T-dimers with lifetimes between 20 and 25 ns. Thus, we suggest that in the cytoplasm, the AG experiences an environment with lower polarity, viscosity, and water content, which results in mostly monomer and π–π dimer populations with lower contribution of the T-dimers that give average lifetimes between 17 and 20 ns. On the other hand, in the nucleoplasm, the AG forms mostly T-dimers that, depending on the local viscosity, give rise to average lifetimes like those observed for the more viscous glycerol/water mixtures. Again, we found lower overall average lifetime values for BT474 in both the cytoplasm and nucleus. Although this different behavior with the other cell lines can be reasonably explained in terms of different local viscosities, polarities, or water quantities, it should be noted that such parameters might differ even between cells of the same origin since it also depends on the cell cytoskeleton and the interaction between cells, extracellular stimuli, and intercellular communication. For instance, the cell viscosity should be considered as a dynamic process that can vary during the different phases of cancer origin and transformation.^47−50^

The observed trend is further confirmed by the emission decays and spectra collected at selected points in the cytoplasm and nucleus (Figures 2G–I and S7A–C, respectively). The emission decays of all the studied cell lines were fit satisfactorily by a biexponential function (excluding the contribution from autofluorescence) giving time components of τ_1_ ∼ 3.7 ns and τ_2_ = 19–23 ns (Table 1). In agreement with the solution studies, we assign these time constants to populations of π–π and T-shaped dimers, respectively. In all the cases, the values of the time constants do not vary significantly for the decays collected in the cytoplasm and in the nucleus. Furthermore, the values are comparable also across the cell lines. While the time constant assigned to the π–π dimers is similar to the one found in solution, the one assigned to the T-shaped dimer is significantly lower (19—21 ns in the cytoplasm and 20–23 in the nucleus) in comparison to the one found in aqueous solutions (25 ns). This behavior suggests that τ_2_ arises from a mixture of two different populations—one that has a lower lifetime, most probably monomers (10–12 ns), and a second one, T-shaped dimers, with a lifetime of ∼25 ns. Notably, the value of τ_2_ is consistently lower in the cytoplasm than in the nucleoplasm, where it approaches the one in solution, which indicates that in the cytoplasm we observe stronger contribution from the AG monomer. Additionally, we found a significant difference in the relative contribution of these populations across the three cell lines. To begin with BT474, the fit to the emission decay collected in the cytoplasm gives 73% relative contribution from the π–π dimers (3.7 ns) and 26% for the mixture of monomers and dimers (20 ns). The ratio changes slightly in favor of the latter, when the decay was recorded in the nucleus increasing to 34%. A similar trend is observed for the contribution of the long-lived population to the decays collected in the cytoplasm for the other cell lines (34 and 32% for MDA-MB231 and MCF7, respectively). However, a notable increase in the contribution of this population is found in the decays for the nucleus. For both cell lines, it becomes the more dominant one reaching 51%, while the value of the related time component also increases to give 22 and 23 ns for MDA-MB231 and MCF7, respectively. This latter increase indicates a significant shift in the monomer–T-shaped dimer equilibrium in favor for the dimer population. The spectra collected within the nucleus are structureless with the emission maximum around 500 nm, while those collected in the cytoplasm are structured and blue-shifted to 480 nm (Figure S7). This observation agrees with the solution experiments, where the π–π dimer emission spectrum is centered at 490 nm, while the T-shaped dimer emission maximum is at 510 nm. This also supports the assignment of the dimer population distribution within the nucleoplasm and cytoplasm.

**Table 1:** Values of Time Constants (τ_*i*_) and Normalized (to 100) Preexponential Factors (*a_i_*) Obtained from the Fit of the Emission Decays of BT474, MDA-MB231, and MCF7 Collected at Representative Points in the Cytoplasm and Nucleus Following Excitation at 390 nm

cytoplasm					nucleus			
	τ_1_ (ns)	*a*_1_ (%)	τ_2_ (ns)	*a*_2_ (%)	τ_1_ (ns)	*a*_1_ (%)	τ_2_ (ns)	*a*_2_ (%)
**BT474**	**3.7**	27	**19**	73	**3.70**	66	**20**	34
**MDA-MB231**	**3.4**	34	**21**	66	**3.70**	49	**22**	51
**MCF7**	**3.9**	32	**20**	68	**3.50**	49	**23**	51

**Figure 3:**
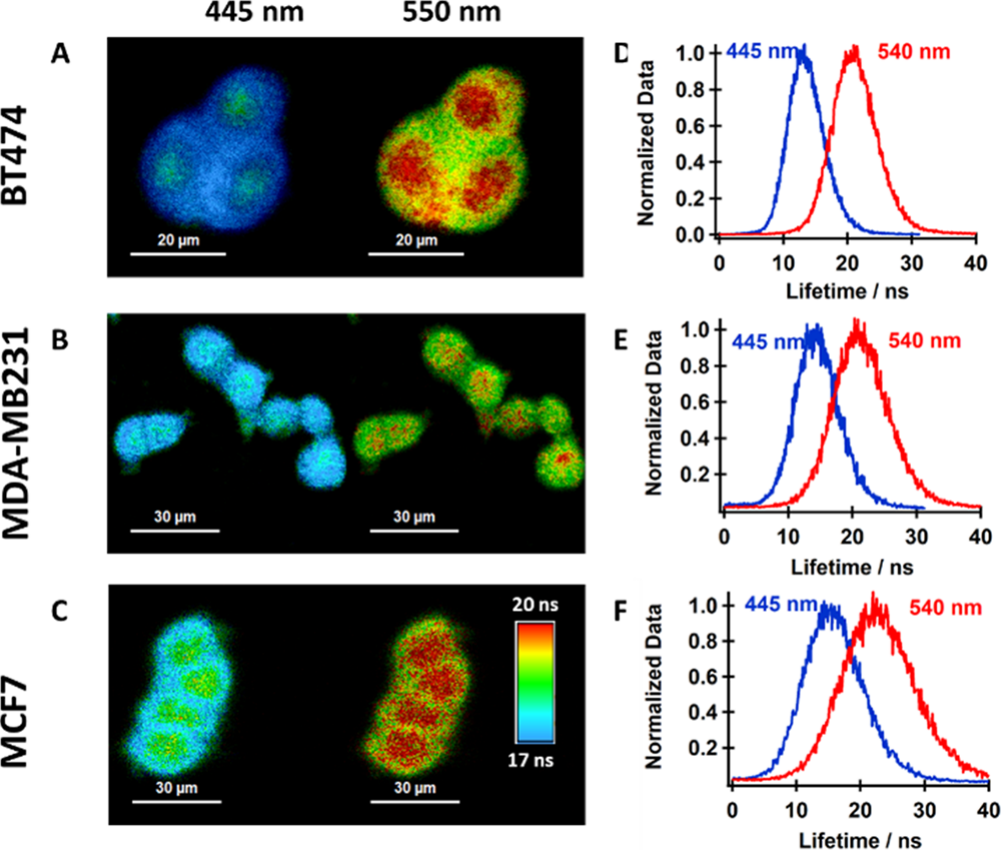
Spectral filtering of the FLIM images (A– C) and the corresponding lifetime distributions (D– F) for the images collected at the two detectors using band-bass filters at the indicated central wavelengths. The scale bars for the FLIM images are 20 μm (A), 30 μm (B), and 30 μm (C).

We also applied spectral filtering to collect the FLIM images for the three cell lines (Figure 3). When the images were collected at the blue side of the emission spectrum (monomer and π–π dimer bands, 445 nm central wavelength), the FLIM images showed stronger contribution of the monomer and π–π dimer populations in the cytoplasm, which gives rise to a broad lifetime histogram centered on ∼14 ns. On the other hand, when the FLIM images were collected at the T-shaped dimer emission band (550 nm central wavelength), we observed that the population with the higher lifetime values (∼23 ns) is located predominantly in the nucleoplasm, while the populations with shorter lifetimes are found mostly in the cytoplasm.

**Figure 4:**
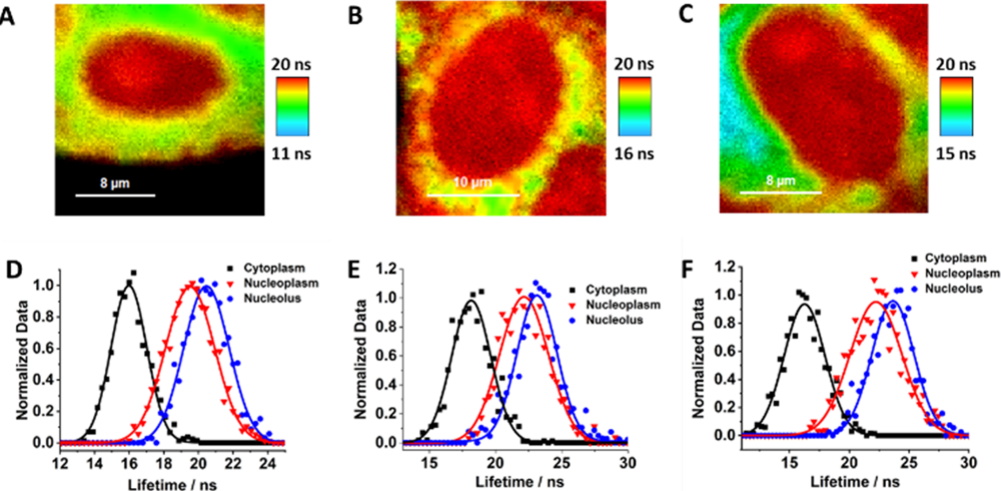
Representative FLIM images of the studied cell lines for BT474 (A), MCF7 (B), and MDA-MB231 (C) along with the respective lifetime distribution histograms for the cytoplasm (black), nucleus (red), and nucleolus (blue) (D–F). The solid lines in D, E, and F represent the Gaussian fit. The scale bars for the FLIM images are 8 μm (A), 10 μm (B), and 8 μm (C).

We examined the FLIM images for the three cell lines following a 24 h incubation period (Figure 4A–C). The images show additional evolution in the relative contribution of the different AG populations. A clear distinction of the cell nucleoli, a nonmembrane-bound nuclear organelle, is observed. While for BT474, we detect mostly single nucleolus (Figure 4A), for MDA-MB231 and MCF7 (Figure 4B,C), we found the presence of several nucleoli. Different numbers of nucleoli have been previously reported for mammalian cell lines with significant variation in their number, shape, and size across different species and cell types.^51^ The lifetime distribution analysis (Figure 4D–F) shows that for the cytoplasm, the histogram shifts consistently towards lower lifetime values for all the cell lines (16, 18.1, and 16.3 ns for BT474, MCF7, and MDA-MB231, respectively), indicating lower contribution of the T-shaped dimer population. On the other hand, the analysis of the nucleoplasm demonstrates two histograms—one associated with the nucleus with slightly lower lifetime values (19.5, 22.1, and 22.2 ns for BT474, MCF7, and MDA-MB231, respectively) and a second one centered on higher values (20.5, 23.2, and 23.6 ns for BT474, MCF7, and MDA-MB231, respectively), associated with the nucleoli that present the higher relative contribution of the T-shaped dimer population.

### Self-Assembly Mechanism

**Figure 5:**
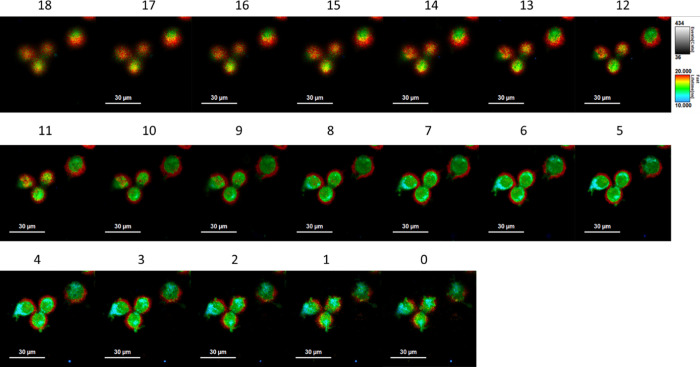
Cross-section FLIM images at 1 μm spacing for the MDA-MB231 cell line following 1 h incubation. Cross-section 18 corresponds to the top-most slice, while 0 is the lowest one. The scale bar is 30 μm.

**Figure 6:**
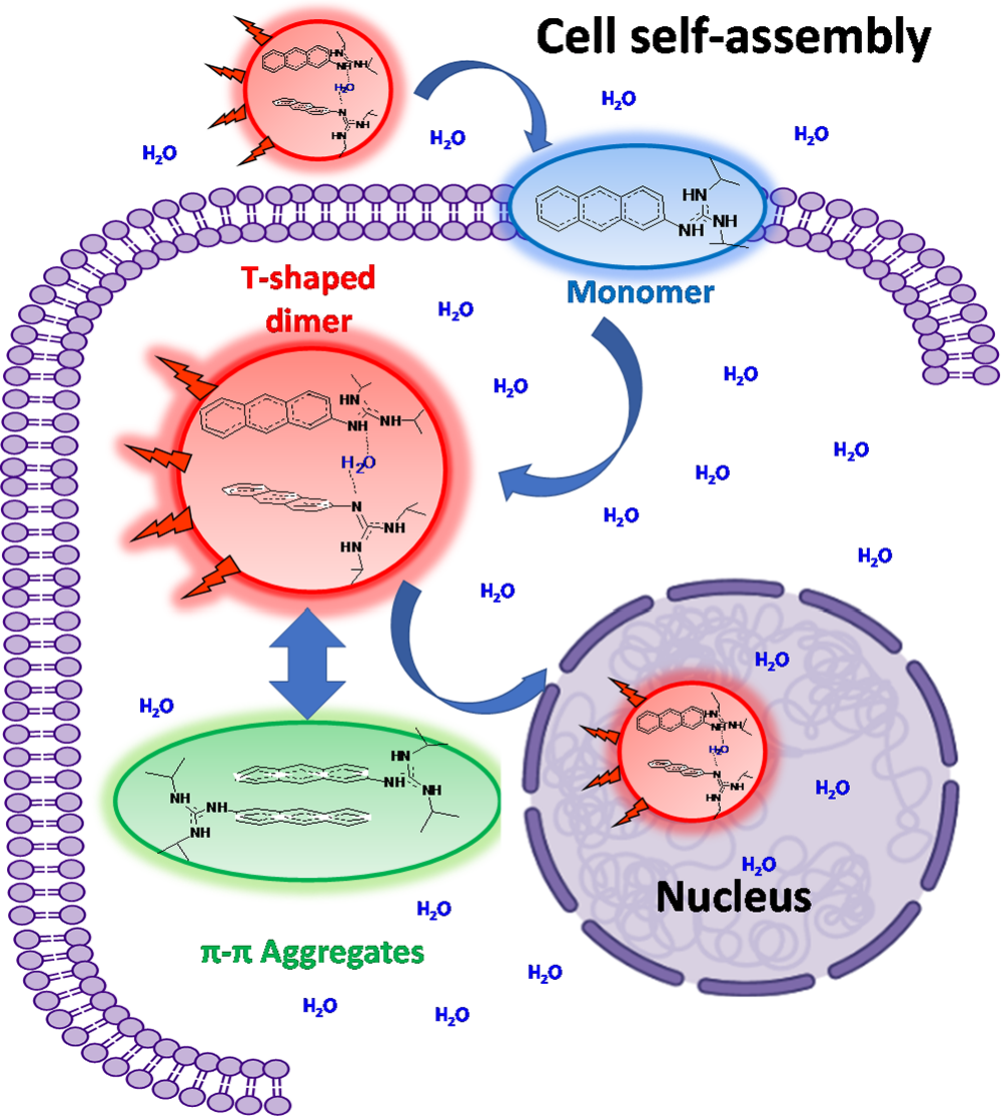
Rationale of the cell self-assembly mechanism of the AG in the cellular environment. The structures are not to scale. The structure of the 1AG monomer is a schematic presentation of the possible monomer forms (charged or neutral) that can cross the membrane (see the text for details).

To further scrutinize the process of self-assembly, we analyzed the FLIM images of MDA-MB231 following a shorter incubation time (1 h). Figure 5 shows the distribution of AG populations in MDA-MB231 at 1 h of incubation at 19 planar slices along the z-axis from top to bottom (step size of 1 μm between slices), while Figure S8 shows the corresponding lifetime distribution histograms. FLIM analysis of the different slices, along with the results observed for the same cell line at 8 h incubation, suggests that the AG molecules reach the cell surface predominantly as T-shaped dimers (∼20 ns, red) where they initially accumulate. This is most evident when studying the top and intermediate cross-sections of the cells (slices 5 to 18) where clear evidence for T-shaped dimer accumulation at the cell membrane can be observed. To cross the hydrophobic membrane, the dimers lose the water molecules in a low viscosity and polarity environment to convert to the more hydrophobic monomeric form (∼10 ns, blue, slices 0–7), which, once in the cytoplasm, recapture water in a polar media and again form T-shaped dimers along with π–π dimers (within 12–18 ns, green). It should be noted that, as an alternative mechanism to cross the membrane, due to the characteristics of the guanidine entity, the monomer form of 1AG might also be present in its charged guanidinium ion form.^52^ Most importantly, no evidence for T-shaped dimer accumulation at the cell membrane is found in the FLIM images for longer incubation times (8 and 24 h), which remarkably present different AG population distributions, with the T-shaped dimer predominantly located in the nucleoplasm and the less emissive π–π dimer found in the cytoplasm (Figure 2B, vide supra). Thus, the formation and distribution of the different AG populations most probably depend on the local viscosity and available water molecules (see the rationale in Figure 6).

Although there is an ongoing debate regarding the microviscosity of the nucleoplasm and cytoplasm, mostly related to the size of the used probe and the crowding and the heterogeneity of the environment, several studies have indicated that their viscosities differ, the former being more viscous than the latter.^47,48,53−57^ A fluorescence correlation spectroscopy study has reported on a nucleoplasm viscosity for HeLa and lung adenocarcinoma (ASTC-a-1) cell lines that were 3 to 4 times larger in comparison to water at 37 °C.^58^ The authors also reported ∼8% lower values for the viscosity of the cytoplasm in comparison to the nucleoplasm. These observations agree with the FLIM images of the cell lines studied here. The more viscous microenvironment of the nucleus allows the AG population to remain mostly in the optimal T-shaped dimer configuration (22–25 ns), while the less viscous cytoplasm facilitates the molecular movement, which in turn gives rise to dimer configurations that are more planar (π–π dimer character) with shorter lifetimes (17–20 ns). These observations are also in agreement with the results for the AG in glycerol/water mixtures (Figure 1B,C), where the increase in the viscosity favors the emission from the T-shape dimer population (∼97% at 50% glycerol/water binary mixtures).

## Conclusions

In summary, we have studied the highly emissive T-shaped dimer formation and distribution in biological media that could be related to variations in the properties of the local environment. FLIM results show that the AG reaches and accumulates at the cell membrane as the T-shaped dimer, which loses the bridging water molecules to give rise to the more hydrophobic monomeric form, which allows its diffusion inside the cell. Once inside the cell the water is recaptured by the AG, and the T-shaped dimer is formed, along with the π–π dimer. Following longer incubation times, the T-shaped dimer is mostly found in the nucleus, probably due to the higher density and viscosity compared to the cytoplasm. The obtained results demonstrate how the intricate balance between the different forms of the AG can be used to probe and report on the properties of the complex cellular local environment. Moreover, the study indicates how the use of a single AIEgen can be advantageous in staining the cell line/s under study without the need of additional chromophores. The application of AIEgens for bioimaging applications is still very scarce due to the inherent difficulty in controlling the self-assembly processes in biological media. In this sense, this work opens up the possibility of using molecules like the AG in a wide range of applications such as viscosity, density, or temperature sensing together with cell cycle or apoptosis monitoring.

## Supporting Information Available

Additional structural (DLS) and spectroscopic (emission spectra and quantum yields) characterization data for 1AG; MTT assay analysis; and additional data on spectral behavior and FLIM results for 1AG in the studied cell lines (PDF)
