# Supplementary material for: Formation of Highly Emissive Anthracene Excimers for Aggregation-Induced Emission/Self-Assembly Directed (Bio)imaging
Source: ACS Appl Mater Interfaces. 2023 Sep 12;15(38):44786–95. doi: 10.1021/acsami.3c10823 (PMC11165449; doi:10.1021/acsami.3c10823)
Supplement: Supplementary file 1 — am3c10823_si_001.pdf [file am3c10823_si_001.pdf]

Supporting Information

Formation of Highly Emissive Anthracene Excimers for Aggregation Induced Emission (AIE) / Self-Assembly Directed (Bio)imaging

Pedro J. Pacheco-Liñán[†], Carlos Alonso-Moreno[†,Ω,∞], Alberto Ocaña[£,¥], Consuelo Ripoll[†,] Elena García-Gil[£], Andrés Garzón-Ruíz[†], Diego Herrera-Ochoa[†], Sofía Blas-Gómez[†], Boiko Cohen[§,]* and Iván Bravo[†,Ω,]*

[†] Unidad nanoDrug. Facultad de Farmacia de Albacete, Universidad de Castilla-La Mancha, 02008 Albacete, Spain.
[¥] Experimental Therapeutics Unit, Hospital clínico San Carlos, IdISSC and CIBERONC, 28040 Madrid, Spain.
[£] Unidad de Investigación del Complejo Hospitalario Universitario de Albacete. Oncología Traslacional, 02008 Albacete, Spain
[Ω] Centro Regional de Investigaciones Biomédicas (CRIB), 02008 Albacete, Spain
[∞] Centro de Innovación en Química Avanzada (ORFEO-CINQA), Universidad de Castilla-La Mancha, 02008 Albacete, Spain
[§] Departamento de Química Física, Facultad de Ciencias Ambientales y Bioquímica, and Instituto de Nanociencia, Nanotecnología y Materiales Moleculares (INAMOL), Universidad de Castilla-La Mancha, Avenida Carlos III, S/N, 45071 Toledo, Spain

*Corresponding Authors: e-mail: ivan.bravo@uclm.es (IB) and boyko.koen@uclm.es (BC)

*** Iván Bravo, Unidad nanoDrug, Facultad de Farmacia de Albacete, Centro Regional de Investigaciones Biomédicas (CRIB), Universidad de Castilla-La Mancha, 02008 Albacete, Spain. E-mail: ivan.bravo@uclm.es**

*** Boiko Cohen, Departamento de Química Física, Facultad de Ciencias Ambientales y Bioquímica, and INAMOL, Universidad de Castilla-La Mancha, Avenida Carlos III, S/N, 45071 Toledo. E-mail: boyko.koen@uclm.es**

Supporting Figures and Tables

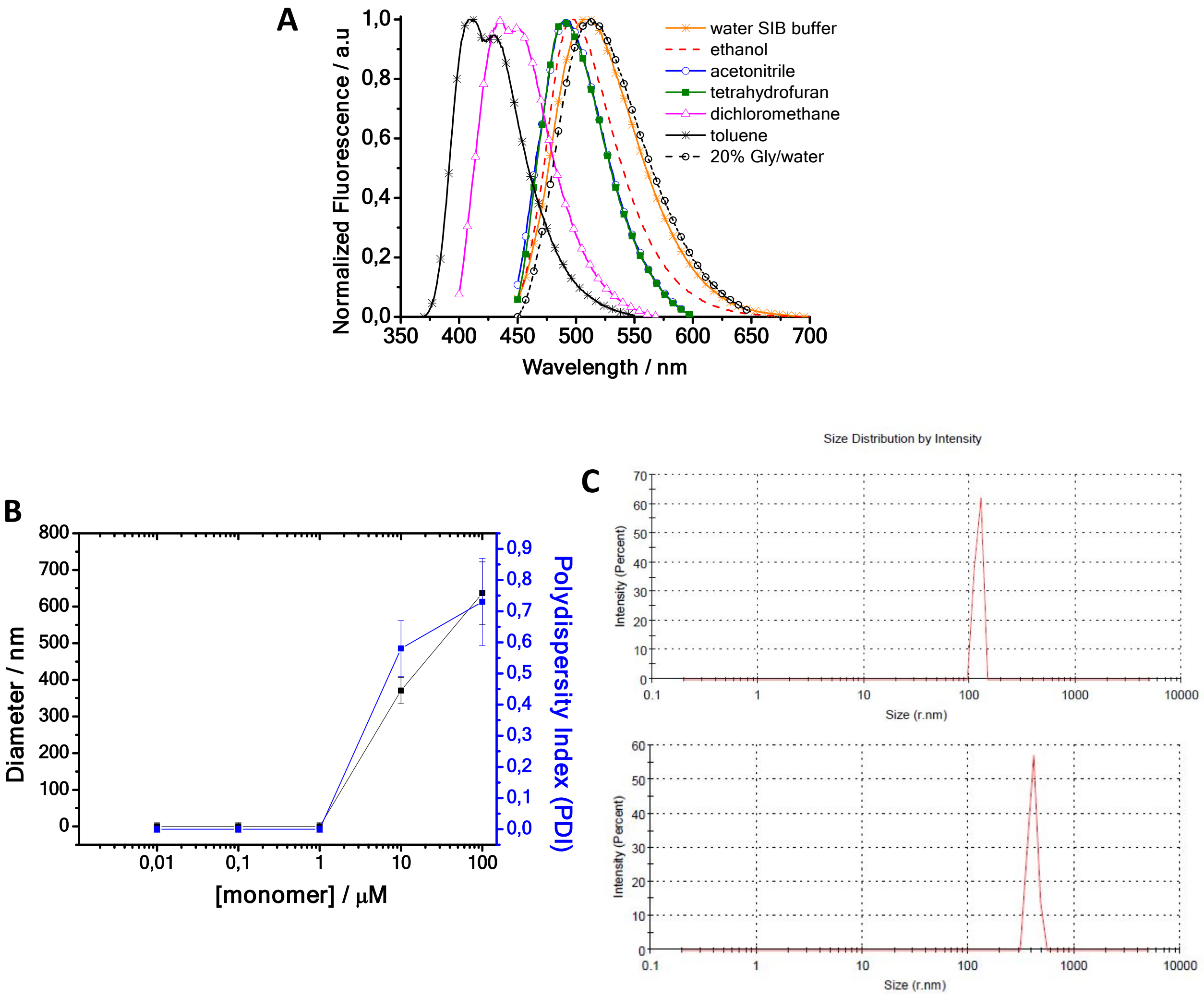

Figure S1. A) Emission spectra of AG (10 µM) in different solvents and B) Dynamic light scattering measurement of size (diameter, nm) and polydispersity index (PDI) of AG at different concentrations. The values represented are the averages of 5 measurements and the error bars are 2σ. C) Some examples of size distribution plots (radius, nm) are shown for 10 and 100 µM monomer concentration.

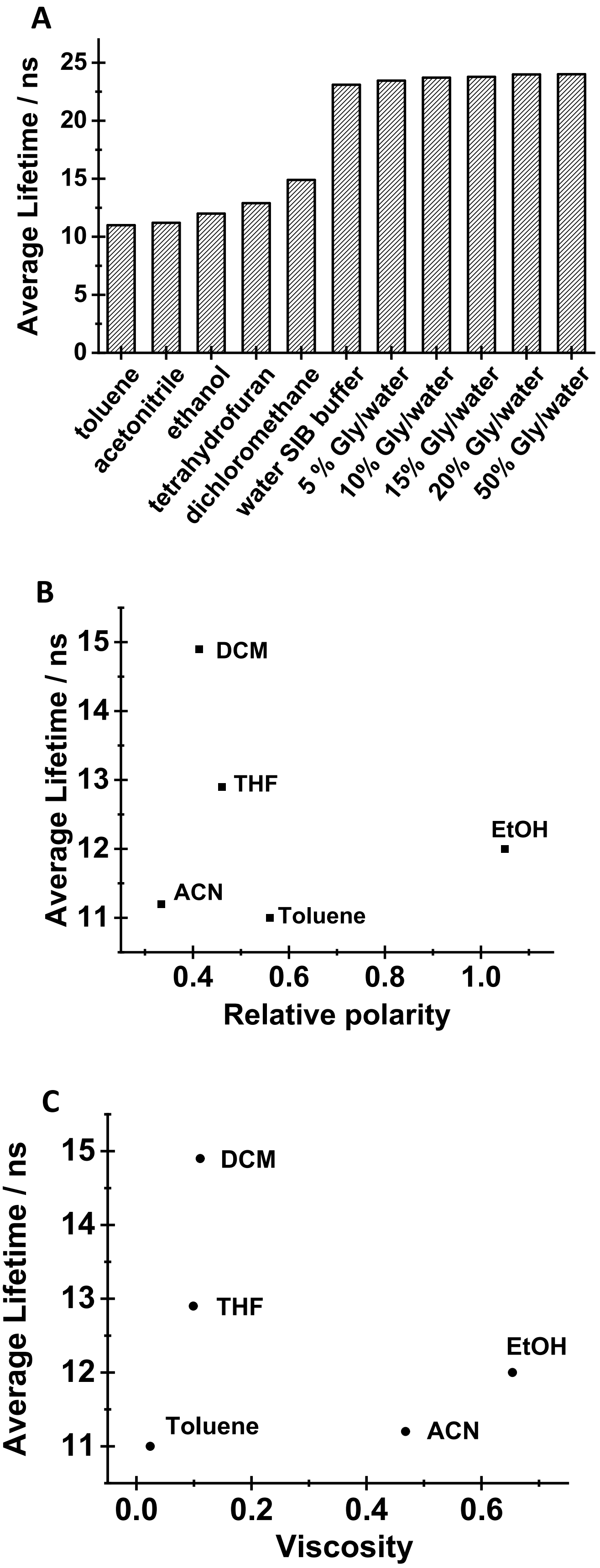

Figure S2. A) Average fluorescence lifetimes of AG (10 μM) in different solutions; B) against the relative polarity (with respect to water); and C) viscosity of organic solvents. Values from obtained from Ref. 1

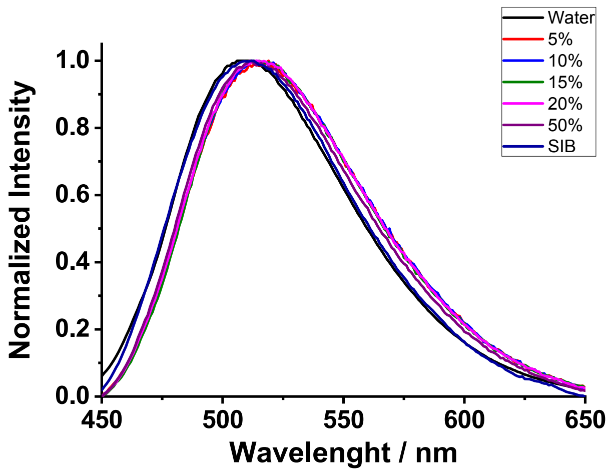

Figure S3. Emission spectra of AG (10 μM) in different glycerol/water mixtures

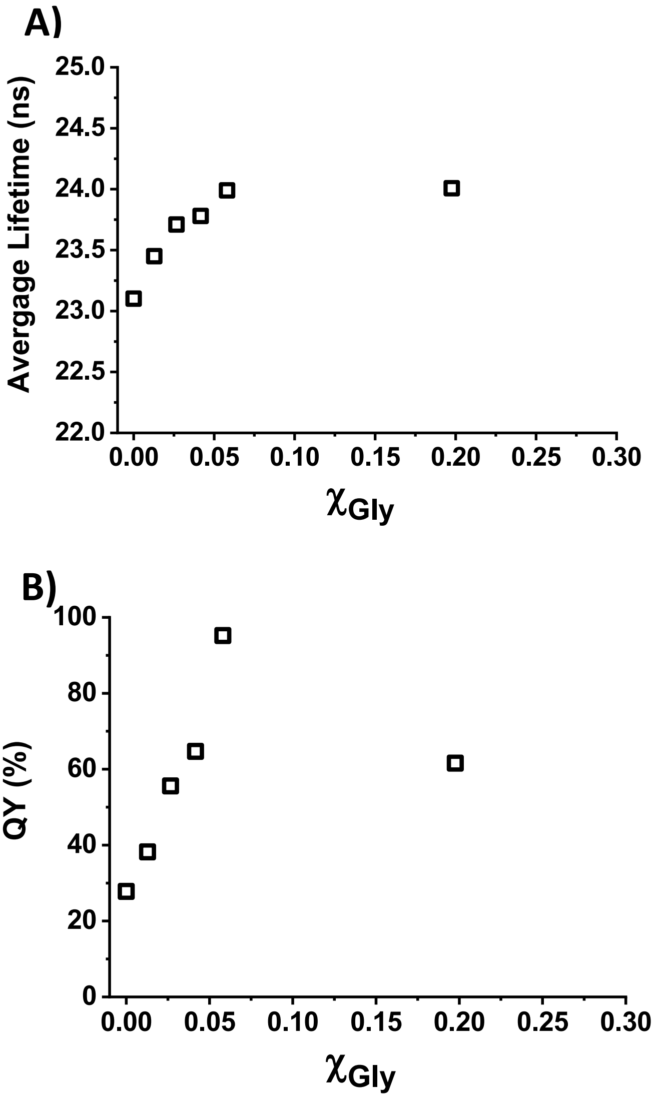

Figure S4. Average fluorescence lifetime (A) and quantum yield (B) values vs molar fraction of glycerol (χ_{Gly}) within SIB and glycerol/water mixtures (up to 50% v/v).

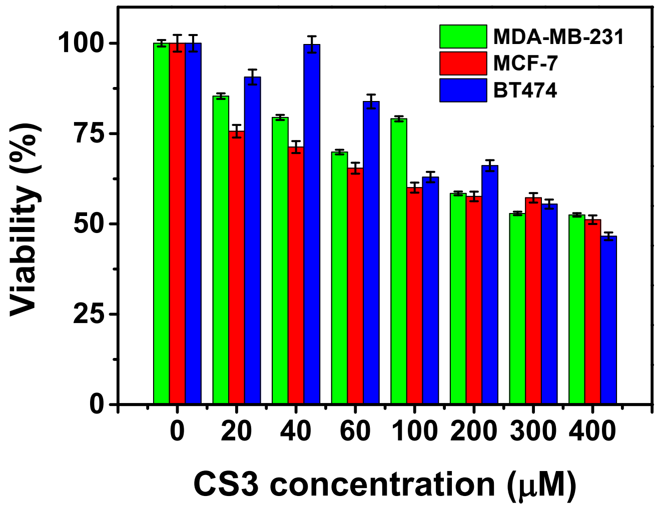

Figure S5. MTT assays of 1AG in the three cell lines studied.

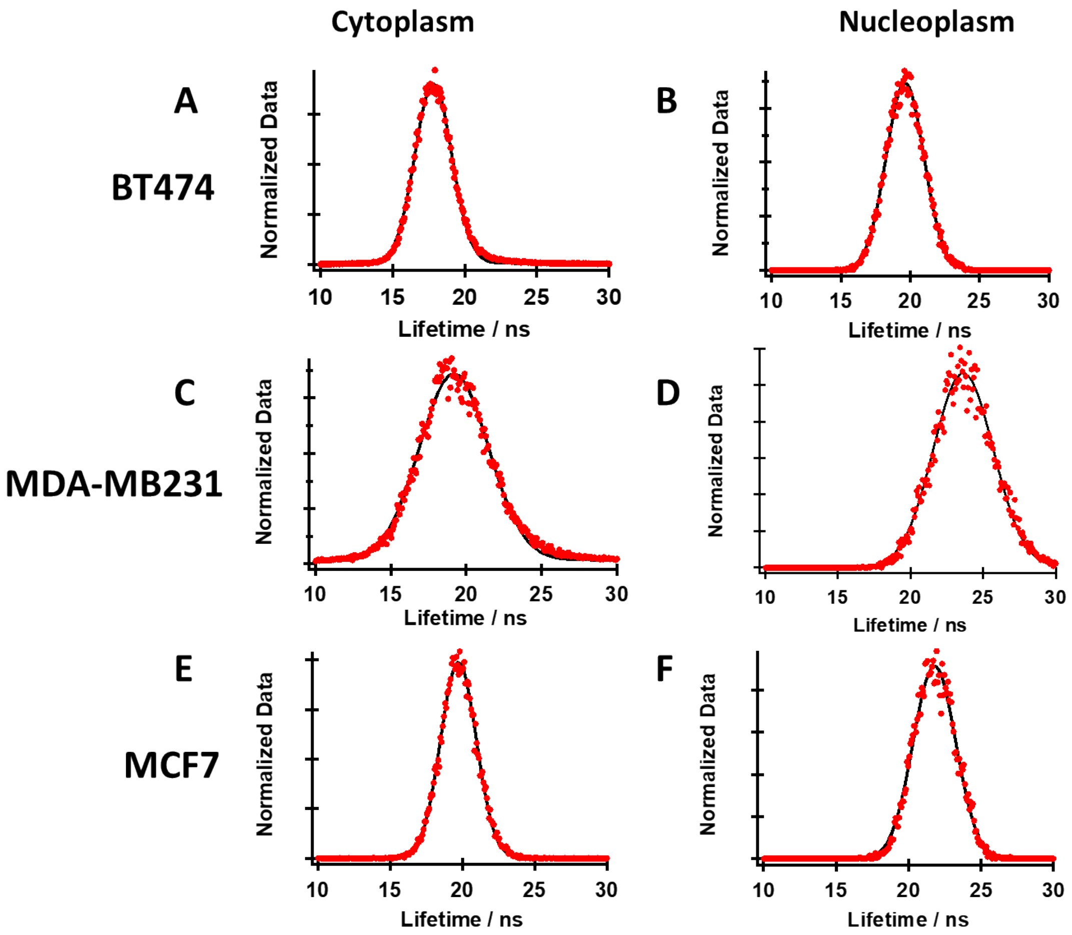

Figure S6. Lifetime distribution histograms for the different cell lines (as indicated) in the figure analyzed selectively in the cytoplasm (A, C and E) and in the nucleoplasm (B, D and F).

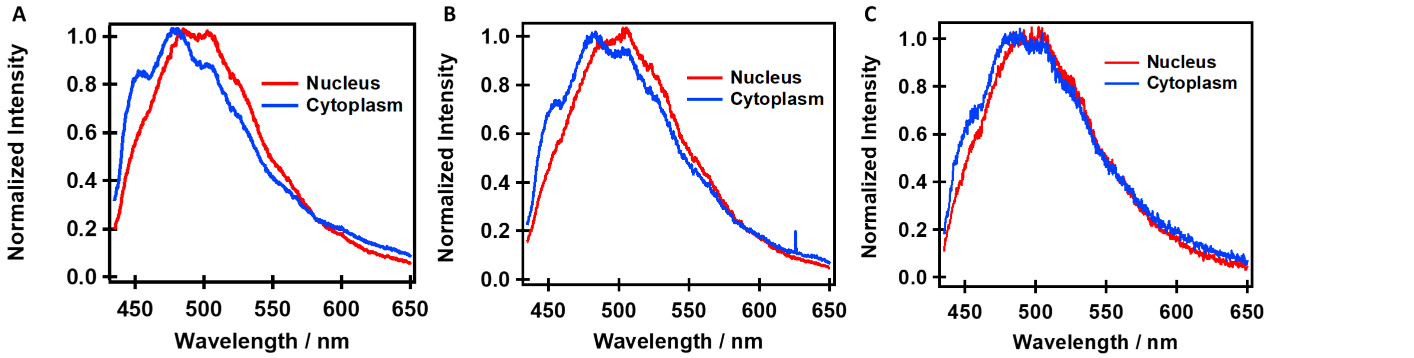

Figure S7. Emission spectra of the studied cell lines: BT474 **(A)**, MDA-MB231 **(B)**, MCF7 **(C)** collected at selected points in the cell nucleus (red) and cytoplasm (blue).

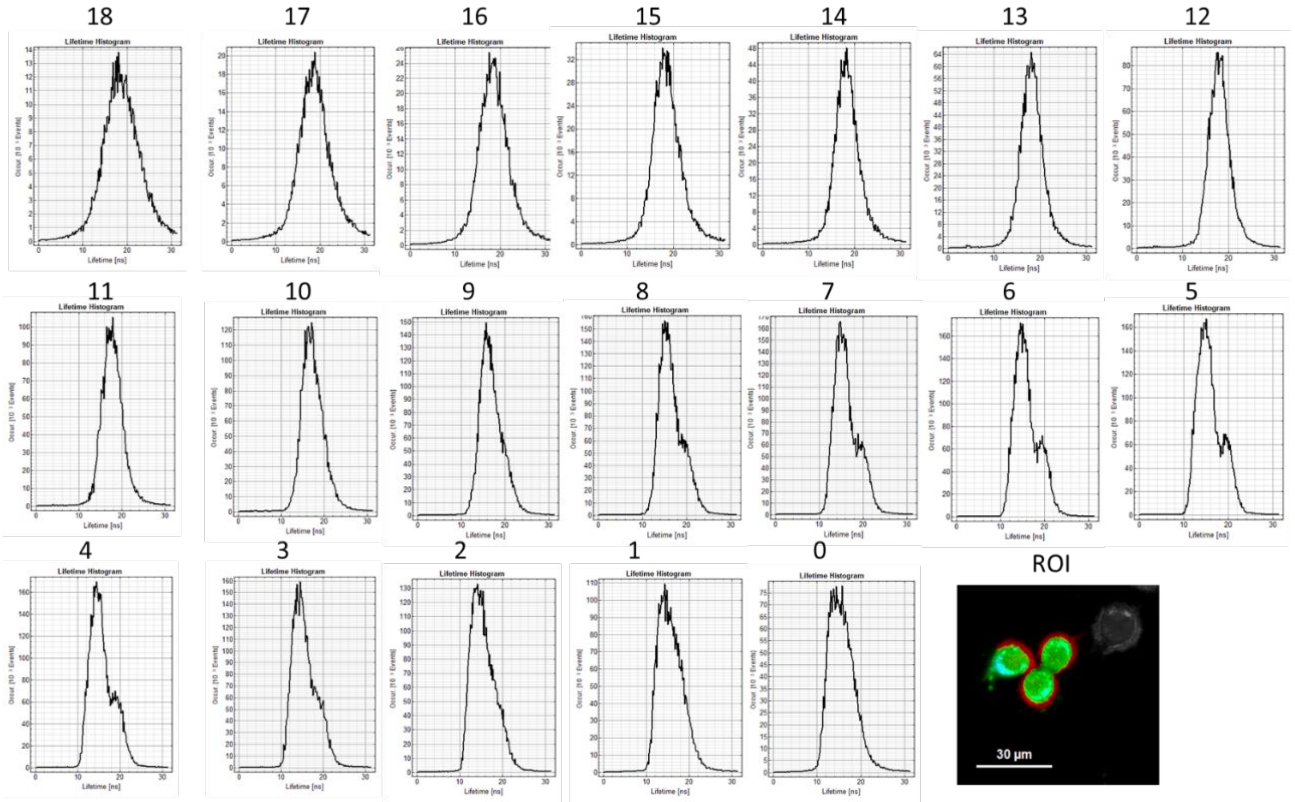

Figure S8. Lifetime distribution histograms for the different FLIM images (Figure 4 in the main text) at 1 μm spacing for MDA-MB231 cell line following 1h incubation. Cross-section 18 corresponds to the top-most slice, while 0 is the lowest one. The image represents the analyzed region of interest (ROI).

Table S1. Quantum yields (QY) measured of different solvents and mixtures (λ_{ex} = 368 nm, λ_{em} = 330 - 650 nm, $\Delta\lambda_{ex}$ = 10 nm, $\Delta\lambda_{em}$ = 0.23 nm)

**Solvent/mixtures**	**QY**
water	27.8%
5% Gly	38.2%
10% Gly	55.6%
15% Gly	64.7%
20% Gly	95.2%
50% Gly	61.6%
Water pH 7.4 SIB buffer	42.0%
Acetonitrile	37.1%
dichloromethane	83.1%
Tetrahydrofurane	69.2%
Ethanol	56.0%

Table S2. Fit parameters for the deconvolution of the overall lifetime distribution histograms of the FLIM images (Figure 2) of the studied breast cancer cell lines. A_i is the relative amplitude, $FWHM_i$ is the full width at half maximum, X_i is the value of the average lifetime and $Area_i$ is the relative area under the curve for the Gaussians following deconvolution of the overall histograms.

	A_1	$FWHM_1$	X_1	$Area_1$	A_2	$FWHM_2$	X_2	$Area_2$	A_3	$FWHM_3$	X_3	$Area_3$
BT474	66±7	1.73±0.22	17.8±2.3	61±5	34±3	2.14±0.21	19.5±1.8	39±2	-	-	-	-
MCF7	-	-	-	-	83±4	1.96±0.19	19.9±1.6	85±3	17±2	1.71±0.17	22.7±1.8	15±2
MDA-MB231	-	-	-	-	90±6	5.08±0.23	19.5±1.6	94±4	10±2	5.16±0.31	23.5±1.6	6±1

Table S3. Fit parameters for the histograms of the FLIM images analyzed only for the nucleoplasm and cytoplasm. $FWHM_i$ is the full width at half maximum and X_i is the value of the average lifetime for the corresponding Gaussians.

	Cytoplasm		Nucleus	
	$FWHM_1$	X_1	$FWHM_2$	X_2
BT474	3.00±0.21	17.8	3.21±0.27	19.6±1.5
MCF7	2.91±0.13	19.7±1.6	3.47±0.32	21.8±1.3
MDA-MB231	5.38±0.31	19.2±1.5	4.88±0.21	23.6±1.7

References

(1) CRC Handbook of Chemistry and Physics: A Ready-Reference of Chemical and Physical Data, 85th Ed Edited by David R. Lide (National Institute of Standards and Technology). CRC Press LLC: Boca Raton, FL. 2004. ISBN 0-8493-0485-7. *J Am Chem Soc* **2005**, *127* (12), 4542. https://doi.org/10.1021/ja041017a.
